# Salt Starvation in the Bromide Treatment of Epilepsy

**Published:** 1902-01

**Authors:** 


### ABSTRACTS.

### SALT STARVATION IN THE BROMIDE TREATMENT OF EPILEPSY.

Dr. A. Pierce Clark, Sonyea, N. Y., in a paper read at the meeting of the Medical Association of Central New York, during its recent meeting at Buffalo, said: Hypochlorisation or salt starvation, as applied to the epileptic dietary, was introduced by Toulouse and Richet in 1899, and has since been successfully used in this country and abroad. Its rationale is that bromine forms a more stable compound in the tissues previously starved by the withdrawal of chlorine, by reducing the amount of table salt ingested.

Under hypochlorisation the daily dosage of bromides may be reduced one-half as well as the chance of bromide intoxication. Then if bromine in the form of bromipin (10 per cent. solution in oil sessanum) is used instead of the bromide salts gastro-intestinal irritation, constipation and bromic ache are abolished more or less entirely.

It is especially recommended in all chronic cases in which bromides, even in high dosage, have failed, and in those altogether intractable to the bromides. In those of poor physique the nutrient emulsion of bromine or bromipin is of advantage combined with the hypochlorisation diet.

Dr. Clark proposed the following sample dietary for carrying out salt starvation treatment in connection with bromides or bromipin: Breakfast—Oatmeal or any other cereal; one pint of milk; bread or toast; potatoes, baked, creamed or boiled; ripe or stewed fruit; eggs, soft boiled, scrambled or poached. Dinner—Vegetable soup strained, bouillon, mutton or chicken broth, oyster soup; beef or mutton, chicken or turkey roasted; tomatoes, vegetable oysters, potatoes, corn, string beans, lettuce, asparagus, spinach; fresh fruits, ice cream or water ices. Supper—One pint milk; milk or dry toast; potatoes creamed or baked; stewed fruit; eggs poached, soft boiled or scrambled; rice.

No salt is to be used on the food or in cooking. Two slices of bread can be eaten with each meal. The fruits should be apples, prunes, peaches, oranges, grape fruit, pears, grapes.

EMULSION BROMIPIN

| | | |
|---|---|---|
| ℞ | Acacia | ℥iii. |
| | Bromipin (Merck) | ℥xvi. |
| | Syrup simplex | ℥iv. |
| | Ol. gaultheria | ʒi. |
| | Aqua, q. s. ad. | ℥xxxii. |
| | Ft. emulsion. | |

Sig.—Two teaspoonfuls, increasing to one ounce as desired, three times a day.

One teaspoonful = Bromine gr. iv.

